# Decreased serotonin transporter immunoreactivity in the human hypothalamic infundibular nucleus of overweight subjects

**DOI:** 10.3389/fnins.2014.00106

**Published:** 2014-05-15

**Authors:** Anke J. Borgers, Karin E. Koopman, Peter H. Bisschop, Mireille J. Serlie, Dick F. Swaab, Eric Fliers, Susanne E. la Fleur, Anneke Alkemade

**Affiliations:** ^1^Department of Endocrinology and Metabolism, Academic Medical Center, University of AmsterdamAmsterdam, Netherlands; ^2^Department of Neuropsychiatric Disorders, Netherlands Institute for Neuroscience, An Institute of the Royal Netherlands Academy of Arts and SciencesAmsterdam, Netherlands; ^3^Cognitive Science Center Amsterdam, University of AmsterdamAmsterdam, Netherlands

**Keywords:** SERT, obesity, hypothalamus, arcuate nucleus, paraventricular nucleus

## Abstract

**Context:** That serotonin plays a role in the regulation of feeding behavior and energy metabolism has been known for a long time. Serotonin transporters (SERT) play a crucial role in serotonin signaling by regulating its availability in the synaptic cleft. The neuroanatomy underlying serotonergic signaling in humans is largely unknown, and until now, SERT immunoreactivity in relation to body weight has not been investigated.

**Objective:** To clarify the distribution of SERT immunoreactivity throughout the human hypothalamus and to compare SERT immunoreactivity in the infundibular nucleus (IFN), the human equivalent of the arcuate nucleus, in lean and overweight subjects.

**Design:** First, we investigated the distribution of serotonin transporters (SERT) over the rostro-caudal axis of six post-mortem hypothalami by means of immunohistochemistry. Second, we estimated SERT immunoreactivity in the IFN of lean and overweight subjects. Lastly, double-labeling of SERT with Neuropeptide Y (NPY) and melanocortin cell populations was performed to further identify cells showing basket-like SERT staining.

**Results:** SERT-immunoreactivity was ubiquitously expressed in fibers throughout the hypothalamus and was the strongest in the IFN. Immunoreactivity in the IFN was lower in overweight subjects (*p* = 0.036). Basket-like staining in the IFN was highly suggestive of synaptic innervation. A very small minority of cells showed SERT double labeling with NPY, agouti-related protein and α–melanocyte stimulating hormone.

**Conclusions:** SERT is ubiquitously expressed in the human hypothalamus. Strong SERT immunoreactivity, was observed in the IFN a region important for appetite regulation, in combination with lower SERT immunoreactivity in the IFN of overweight and obese subjects, may point toward a role for hypothalamic SERT in human obesity.

## Introduction

The brain serotonin (5-HT) system is known to be involved in the regulation of food intake and body weight. Several animal experimental studies manipulating endogenous serotonin synthesis, availability and metabolism, have made it clear that there is a negative relationship between the level of brain serotonin and food intake, in which increasing serotonin (by inhibiting reuptake or activating serotonin receptors post-synaptically) inhibits food intake (Lam et al., 2010). In addition, over the last few years evidence has accumulated on the central serotonin system's involvement in peripheral glucose metabolism (Lam and Heisler, 2007). A more recent study reports that serotonin transporter (SERT) knockout mice are obese and present pre-diabetic symptoms such as glucose intolerance and insulin resistance (Chen et al., 2012).

A key brain area involved in the regulation of feeding behavior and in peripheral glucose metabolism is the hypothalamus. There are a number of anatomically distinct nuclei within the hypothalamus that are involved in these functions and that are closely interrelated (Saper et al., 2002). A central role in this is played by the arcuate nucleus (ARC), or infundibular nucleus (IFN) as it is referred to in humans (Swaab, 2003). Several neuropeptides related to both feeding behavior and glucose metabolism have been localized in the ARC/IFN. Like has been shown for rodents, Neuropeptide Y (NPY), agouti-related protein (AGRP) and α-melanocyte stimulating hormone (αMSH) are localized in the human IFN (Goldstone et al., 2002; Alkemade et al., 2012; Saderi et al., 2012). When these neuropeptides are injected into the rodent brain, NPY and AGRP increase feeding, reduce insulin sensitivity, and increase glucose production. On the other hand, αMSH decreases food intake and increases insulin action, thus reducing glucose production and enhancing glucose uptake (for review: Arble and Sandoval, 2013). More recently we investigated the expression of these neuropeptides in the human IFN in relation to body mass index (BMI), and type 2 diabetes (Alkemade et al., 2012). We found that AGRP expression showed a U-shaped correlation with BMI, and that NPY expression was lower in overweight and obese subjects, whereas αMSH revealed no relation to BMI.

Although it is clear that serotonin has an effect on feeding behavior and glucose metabolism in the rodent hypothalamus, data on human hypothalamus are scarce. What has been shown in humans is that polymorphisms related to serotonergic signaling are associated with BMI (Heisler and Tecott, 1999; Sookoian et al., 2008), however this does not provide evidence for hypothalamic serotonin as serotonin signals throughout the body and also widely throughout the brain. We did recently show that hypercaloric high fat high sugar snacking reduces diencephalic SERT (Koopman et al., 2013). Although diencephalon contains hypothalamus, it remains to be determined whether SERT containing cells/fibers can be identified in the human IFN and other hypothalamic nuclei. If identified, the question arises which individual nuclei contain the densest SERT staining and how SERT staining in the IFN relates to neuropeptides known to be involved in energy metabolism.

In this paper we determined the distribution of SERT immunoreactivity in the human hypothalamus using a post-mortem approach. In addition, we compared SERT staining in the IFN of subjects with a BMI < 25 to that of subjects with a BMI ≥ 25 kg/m2. Finally, we describe immunofluorescent double-labeling of SERT with the IFN neuropeptides NPY, AgRP, and αMSH to identify cell types showing basket-like staining of SERT-immunoreactive fibers, suggestive of synaptic innervation.

## Materials and methods

### Subjects

For *Experiment 1* we investigated the distribution of SERT using systematic sampling over the entire rostro-caudal axis of the hypothalamus of 6 subjects (3 male) without neurological or psychiatric disease ranging in age between 67 and 86 years. Clinicopathological and relevant medication data are presented in Table 1.

**Table 1:** **Brain material experiment 1**.

**Subject**	**Sex**	**Age**	**PMD**	**Fix**	**Cause of death, clinical diagnoses**
99101	M	69	19	41	Respiratory insufficiency, infarction distal brainstem, alcohol abuse, hypertension
00007	M	85	15	35	Myocardial infarction, bladder carcinoma
03054	M	67	4.5	50	Cardiogenic shock, multi organ failure, type 2 diabetes (insulin, metformin), COPD
98056	F	83	5	41	Respiratory insufficiency, colon carcinoma, type 2 diabetes (no data available on medication), cataract, arthrosis
95016	F	86	13.5	30	Decompensatio cordis, type 2 diabetes (tolbutamide), angina pectoris, nephropathy, retinopathy
01005	F	76	20	36	Respiratory insufficiency, Non-Hodgkin lymphoma, hypertension, basal cell carcinoma

Fix, duration of fixation in days; PMD, post-mortem delay in hours.

For *Experiment 2* in which we related SERT staining in the IFN to BMI, we studied post-mortem hypothalamic tissue of 11 overweight and obese (6 male, median BMI 30.5 (range: 25.0–39.5) kg/m^2^; median age 76 (range: 65–100) years) with 12 non-obese (5 male, median BMI 20.1 (range15.2–24.2) kg/m^2^; median age 64 (range: 50–92) years) subjects. Clinicopathological and relevant medication data have been published earlier (Alkemade et al., 2012), and are presented in the Table 2.

**Table 2:** **Brain material experiment 2**.

**Subject**	**Sex**	**Age**	**PMD**	**Fix**	**BMI**	**Cause of death, clinical diagnoses**
09102	F	92	7	51	15.2	Cachexia, rectal carcinoma, old CVA
98072	M	79	17	31	17.0	Pneumonia, COPD, CVA, renal insufficiency
98091	F	50	41	72	18.8	Respiratory insufficiency, pancoast tumor, metastasized
98027	M	54	8	59	18.9	Bronchopneumonia, sepsis, hepatocellular carcinoma, pneumococcus infection
09075	M	88	7	44	19.6	Respiratory insufficiency, COPD, cardiac insufficiency, PTCA, adenocarcinoma
91205	F	65	9:30	10	20.0	Cardiac failure, mammary carcinoma
10008	M	81	4:50	53	20.3	Angina pectoris, hypercholesterolemia, adenocarcinoma, arthritis
08052	F	62	8	73	21.0	Respiratory insufficiency, renal cell carcinoma, metastasized
98161	F	60	8	87	22.9	Pneumonia, ovarian carcinoma, metastasized
98030	M	63	2:30	108	23.8	Respiratory insufficiency, squamous cell carcinoma of the oropharynx, metastasized
98095	F	75	ND	63	24.0	Respiratory insufficiency, pneumonia, COPD, atrial fibrillation, ischemic heart disease mycosis fungoides
10039	F	60	7	88	24.2	Cardiac insufficiency, angina pectoris, old CVA
97100	M	76	19	133	25.0	Septic syndrome after aorta bifurcation prosthesis
10013	M	70	6	68	26.0	Pulmonary embolism, adenocarcinoma
08054	F	92	7	67	27.4	Heart failure, melanoma, type 2 diabetes (no data available on medication), cholecystolithiasis, hepatic steatosis, ileus, cataract
09039	M	82	13	38	27.7	Heart failure, angina pectoris, prostate carcinoma, cataract
01021	M	56	5:30	35	27.8	Myocardial infarction, unsuccessful resuscitation, atrial fibrillation, heart failure
97146	F	100	24	62	30.5	Pneumonia, tibia, and fibula fracture
01005	F	76	19:30	36	32.0	Respiratory insufficiency, Non-hodgkin lymphoma
97065	F	76	14:30	27	32.3	Renal insufficiency, heart failure
09095	F	71	7	53	32.9	Renal insufficiency, hypertensive nephropathy, CVA
97060	M	65	59:30	36	34.7	Myocardial infarction, heart failure, type 2 diabetes (tolbutamide, metformin)
00072	M	78	18	45	39.5	Renal insufficiency, atrial fibrillation, heart failure, dehydration

*Fix, duration of fixation in days; PMD, post-mortem delay in hours; BMI, body mass index in kg/m^2^*.

In experiment 3 we aimed to identify the immunocytochemical nature of the cells showing basket-like SERT immunoreactivity, using brain material of the subjects described in Experiment 1. All brain material was obtained from The Netherlands Brain Bank at The Netherlands Institute for Neuroscience (director Dr. I. Huitinga) in accordance with the formal permissions for brain autopsy and for the use of human brain material and clinical information for research purposes.

### Histology

Brains were dissected at autopsy and the hypothalamus was fixed in 10% phosphate-buffered formalin at room temperature (RT) for 1–2 months. After dehydration in graded ethanol series, tissues were cleared in toluene, and embedded in paraffin. Coronal serial sections (6 μm) were cut over the entire rostro-caudal axis of the hypothalamus. For anatomical orientation, every 100th section was collected and mounted on chrome alum-gelatine coated glass slides and subsequently dried for 2 days at 37°C, followed by Nissl staining.

### Antibody characterization

Mouse monoclonal anti-human SERT antibody was purchased from Millipore, MAb Technologies Inc. (Stone Mountain, GA; catalog no Mab5618). Antibody specificity has been reported before and was supported using Western blotting (Bauman et al., 2000; Ramsey and Defelice, 2002; Serafeim et al., 2002; Henry et al., 2003). Rabbit polyclonal anti-human AGRP antibody was obtained from Phoenix Pharmaceuticals (Belmont, CA; catalog no. H-003-53). AGRP staining disappeared after pre-adsorption with AGRP and was not affected by cross adsorption using the NPY peptide (Goldstone et al., 2002). The αMSH antibody was raised against the αMSH C-terminal, which is modified in αMSH free acid, and absent in ACTH, minimizing cross reaction with other POMC products. Staining was abolished after pre-adsorption with the αMSH peptide (Elias et al., 1998).

### Immunohistochemistry

Histological and immunocytochemical processing was performed as described previously, with some minor modifications (Alkemade et al., 2012). For SERT-immunohistochemistry, a series of coronal sections at 100-section intervals over the entire rostro-caudal axis of the hypothalamus was mounted on Superfrost plus slides (Menzel Glaser, Germany) and dried for at least 2 days at 37°C. This resulted in 6–12 sections per subject. Next, antigen retrieval was performed using microwave treatment (Shi et al., 1997) and sections were stained using the avidine biotinylated complex method (Hsu et al., 1981), according to the following protocol: Sections were deparaffinised in xylene and rehydrated through graded ethanol series. After rinsing in distilled water, the sections were washed in TBS (pH 7.6) and antigen retrieval was performed using microwave treatment (10 min 700 W) in TBS (pH 7.6). After adjustment to RT, sections were incubated in the primary antibody diluted 1:5000 in SUMI [supermix, 0.05 M Tris, 0.15 M NaCl, 0.5% Triton X-100 (Sigma, Zwijndrecht, The Netherlands), and 0.25% gelatine (Merck Darmstadt, Germany) (pH 7.6)] overnight at 4°C. The slides were rinsed in TBS (pH 7.6, 3 × 5 min) and incubated for 1 h at RT in the second antibody (biotinylated horse anti-mouse 1:400 in SUMI; Vector Laboratories, Burlingame, CA). After rinsing in TBS (pH 7.6, 3 × 5 min), the sections were incubated 1 h at RT in avidine biotinylated complex (1:800 in SUMI; Vector Laboratories, Burlingame, CA) and subsequently rinsed in TBS (pH 7.6, 3 × 5 min). Finally, sections were incubated in 0.5 mg/ml 3,3'-diaminobenzidine (Sigma) in TBS containing 0.2% ammonium nickel sulphate (BDH; Brunschwig, Amsterdam, The Netherlands) and 0.01% H_2_O_2_ (Merck) for approximately 15 min. The reaction was stopped in distilled water. The sections were dehydrated in ascending series of ethanol, cleared in xylene, and coverslipped using Entellan (Merck, Darmstadt, Germany).

### Double-labeling by immunohistochemistry

To identify cell types showing basket-like staining of SERT-immunoreactive fibers in the IFN, we performed immunofluorescent double staining of respectively SERT with αMSH (1:20000), NPY (1:1000), and AGRP (1:3000) in hypothalamic sections containing the IFN of all 6 subjects.

After overnight primary antibody incubation at 4°C the slides were rinsed in TBS (pH 7.6, 3 × 5 min) and incubated in the secondary antibodies (biotinylated horse anti-mouse 1:400 in SUMI; Vector laboratories, Burlingame, CA) for 1 h at RT. Following rinsing in TBS (pH 7.6, 3 × 5 min), the sections were incubated 1 h at RT in avidine biotinylated complex (1:800 in SUMI; Vector Laboratories, Burlingame, CA), subsequently rinsed in TBS (pH 7.6, 3 × 5 min) and incubated in biotinylated tyramide (1:750 in SUMI, 0.01% H_2_O_2_ (Merck, Darmstadt, Germany)) for 15 min at RT followed by rinsing in TBS (pH 7.6, 3 × 5 min). SERT was detected in green by streptavidin-Alexa488 (1:1000; Invitrogen, Eugene, Or). The other peptides were visualized in red by respectively anti-rabbit Alexa594 (1:1000; Invitrogen, Eugene, Or) for NPY or AGRP and anti-sheep Alexa594 (1:1000; Invitrogen, Eugene, Or) for αMSH. This fluorochrome-conjugated antibody incubation was performed for 1 h at RT, followed by overnight incubation at 4°C.

Vectashield with DAPI (Vector laboratories, Inc, Burlingame, CA) was used for nuclear staining and cover slipping. The sections were stored under dark conditions at 4°C until further analysis. Colocalization was assessed by visual inspection.

### Quantitative analysis

SERT immunoreactivity was quantified using an unbiased masking procedure of the 3,3'-diaminobenzidine-Ni precipitate (Alkemade et al., 2012). For quantification of the immunoreactive signal, gray values of the DAB-Ni precipitate in the IFN were analyzed by computer-assisted densitometry using Image pro (Media Cybernetics, Silver Spring) and software developed at the Netherlands Institute for Neuroscience. Every 100th section containing the IFN was analyzed and estimates of the immunoreactivity were made by averaging the signal density of the 3 sections that showed the highest signal, resulting in arbitrary units (a.u.) (Alkemade et al., 2003).

### Statistical analysis

The data of the second experiment were analyzed with SPSS for Windows, version 19.0 (SPSS Inc. Chicago, Illinois, USA). Group differences in numerical variables were evaluated using the Mann-Whitney *U*-test for not normally distributed parameters. A stepwise linear regression analysis was performed to investigate the effects of possible confounding factors. The statistical significance level for all analyses was set at *p* < 0.05 (two-sided).

## Results

**Figure 1:**
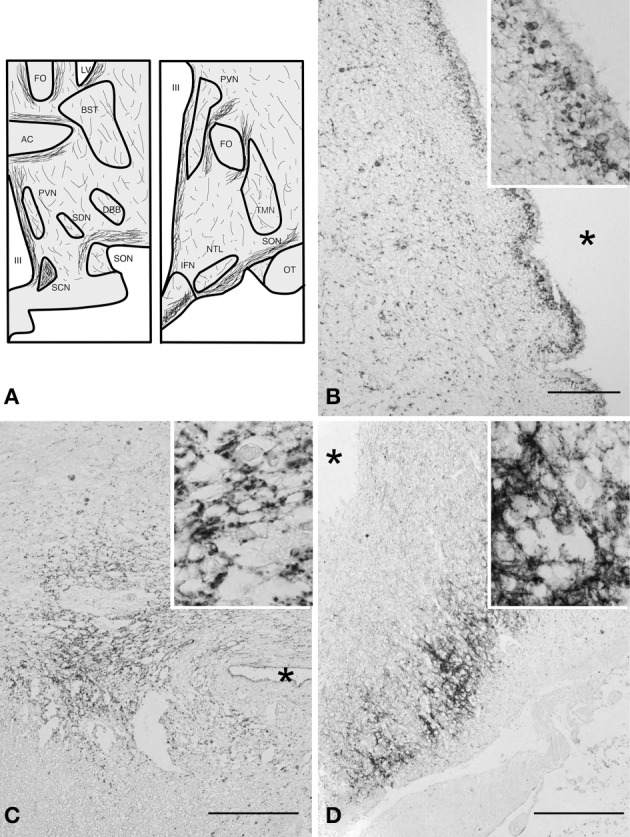
**(A)** Schematic illustration of the distribution of immunoreactive serotonin transporter (SERT) fibers in coronal sections (rostral and caudal) of the human hypothalamus. Abbreviations: AC, anterior commissure; BST, bed nucleus of the striae terminalis; DBB, diagonal band of Broca; FO, fornix; IFN, infundibular nucleus; LV, lateral ventricle; NTL, lateral tuberal nucleus; OT, optic tract; PVN, paraventricular nucleus; SCN, suprachiasmatic nucleus; SDN, sexually dimorphic nucleus; SON, supraoptic nucleus; TMN, tuberomamillary nucleus; III, third ventricle. **(B)** SERT staining, ependymal layer at the level of the PVN bar = 100 μm, **(C)** suprachiasmatic nucleus, bar = 250 μm, **(D)** infundibular nucleus, bar = 500 μm. ^*^Indicates the third ventricle. High power inserts are from the same anatomical structures.

Many SERT-immunoreactive fibers and few scattered SERT-positive basket cells were found throughout the entire hypothalamus, except in the white matter tracts (Figure 1). Basket-like staining was defined as aggregation of staining surrounding neuronal cell bodies, creating a basket-like appearance, suggestive of synaptic innervation. The general distribution of SERT was comparable in all subjects studied (Figure 1A). A strong inter-individual variation in staining intensity was observed. SERT positive fibers were present throughout the entire hypothalamus, with a denser network of SERT-immunoreactive fibers in the perifornical area and in close proximity to the anterior commissure. A plexus along the ependyma of the third ventricle wall also showed strong SERT-immunoreactivity (Figure 1B). In addition, the highest fiber density was observed in the IFN, and the suprachiasmatic nucleus, which is the central biological clock of the human hypothalamus (Figures 1C,D). In these areas, cell bodies and capillaries were directly surrounded by clusters of SERT-immunoreactive fibers, highly suggestive of SERT-positive nerve endings in contact with SERT-negative perikarya and capillaries (high power inserts of Figures 1C,D). The supraoptic nucleus, paraventricular nucleus, lateral tuberal nucleus, and tuberomamillary nucleus contained relatively small numbers of SERT-immunoreactive fibers. No differences were observed between males and females.

**Figure 2:**
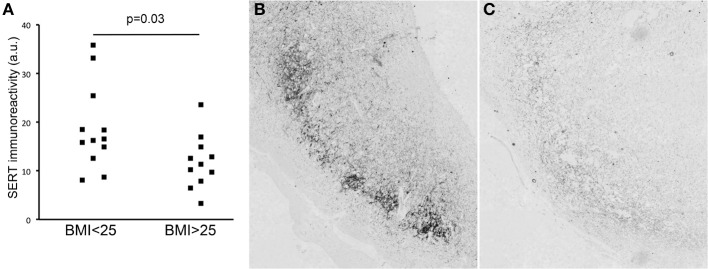
**(A)** SERT immunoreactivity in lean vs. overweight subjects. Illustration of SERT staining in the infundibular nucleus of a lean **(B)** and an obese **(C)** subject.

SERT-staining was observed in the IFN of all subjects. IFN SERT-immunoreactivity was lower in overweight subjects than in non-overweight subjects (*p* = 0.036) (Figure 2A). An example of the difference between overweight and non-overweight subjects is illustrated in Figures 2B,C. A stepwise linear regression analysis performed for the factors age, sex, fixation duration, and post-mortem delay did not reveal any significant results.

**Figure 3:**
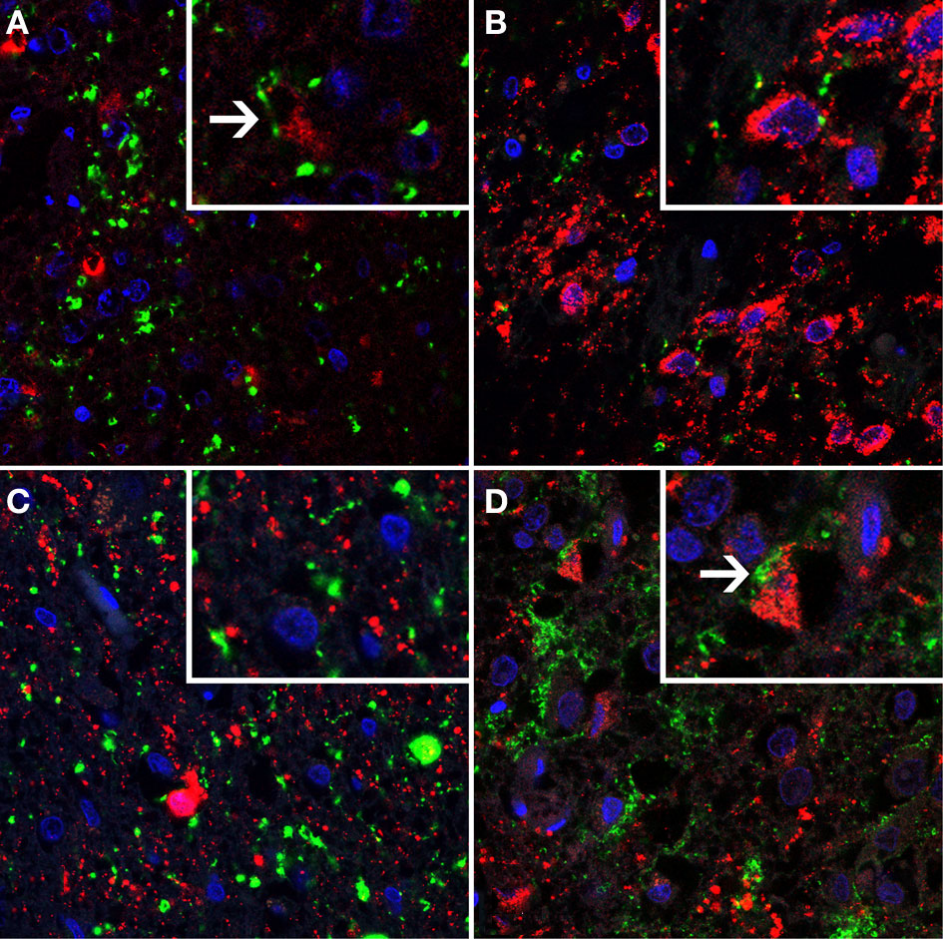
**A series of immunofluorescent photomicrographs showing serotonin transporter (SERT)- immunostaining (shown in green) combined with markers for major cell types (shown in red) of suprachiasmatic nucleus (SCN) and infundibular nucleus (IFN).** Cell nuclei are shown in blue. SERT immunofluorescent staining (green) combined with **(A)** arginine vasopressin (AVP, red) in the SCN, high power insert shows SERT staining in close proximity of AVP-positive neurons which was observed in a very small minority of cells; **(B)** Vasointestinal peptide (VIP, red), high power insert illustrates the absence of SERT immunoreactivity surrounding VIP-positive neurons; **(C)** Neuropeptide Y (NPY, red), and **(D)** αMelanocyte stimulating hormone (αMSH, red), note the SERT staining surrounding the αMSH positive neurons.

**Figure 4:**
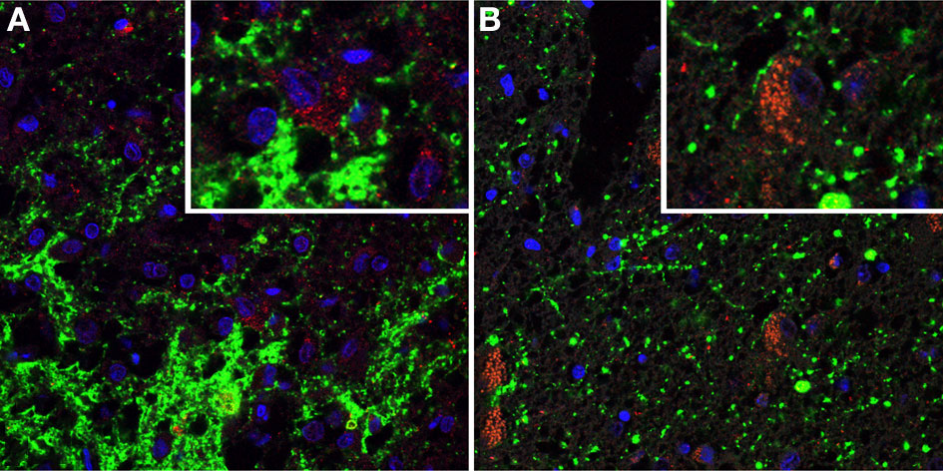
**Immunofluorescent photomicrographs showing serotonin transporter (SERT)-immunostaining (shown in green) combined with immunostaining for Agouti Related Peptide (AGRP) (shown in red).** Cell nuclei are shown in blue. **(A)** The IFN of subject 00007. Note heavily stained SERT-positive fibers. The high power insert shows basket-like SERT staining surrounding neurons which are not immunoreactive for AGRP **(B)** The IFN of subject 01005. The high power insert shows an AGRP immunoreactive cell surrounded by SERT-positive fiber, suggesting nerve ending.

To further characterize the cell types showing basket-like staining of SERT-immunoreactive fibers in the IFN, we performed immunofluorescent double staining of SERT with NPY, AGRP, or αMSH on sections containing the IFN. SERT-immunoreactive fibers were present at all levels in all studied subjects. Many single-labeled cells expressing NPY, AGRP and αMSH-immunoreactive cells were found in the IFN in all subjects. A very small minority of neurons immunoreactive for αMSH or AGRP showed basket-like SERT staining. As SERT showed no clear colocalization with NPY, neurons showing basket-like SERT staining remained largely unidentified (Figures 3, 4). Of note, the labeling of SERT in the IFN was pre-dominantly localized within the IFN, somewhat lateral to the NPY, AGRP-, and αMSH-immunoreactive cells. A number of these cells appeared to be strongly activated, as indicated by the presence of two nucleoli. Together, our results indicate that SERT axons project to a minority of αMSH and AGRP neurons, as well as to currently unidentified subgroups of neurons in the IFN.

## Discussion

We showed that SERT protein is extensively expressed in the human hypothalamus. Moreover, one of the nuclei most heavily innervated was the SCN, which is in agreement with the distribution of SERT immunoreactivity reported in rodents (Legutko and Gannon, 2001) as well as in human and non-human primates (Moore and Speh, 2004; Emiliano et al., 2007). Interestingly, when analysing SERT protein in post-mortem sections of the IFN of overweight and obese subjects and comparing them to lean individuals, the amount of SERT protein was clearly reduced in obesity. This is in line with a large number of knockout and transgenic mouse studies, which, collectively, show an inverse relationship between brain serotonin signaling and food intake, as well as with pharmacological studies targeting serotonin signaling in rodents (for review: Lam et al., 2010). We cannot exclude that BMI in these subjects was influenced by severe illness, including malignancies such as adenocarcinomas. In addition, some subjects received antidiabetic medication. Immunoreactivity for serotonin has been reported before in the rodent ARC, where stained fibers were found in the immediate vicinity of capillaries and neurons (Warembourg and Poulain, 1985). Surprisingly, our double-labeling study indicates that these SERT immunoreactive fibers in the IFN only partly belonged to AGRP or αMSH basket cells. Interestingly, the majority of SERT fibers was located lateral to the neuronal populations expressing peptides NPY/AGRP and αMSH which could point to a role for serotonin in a different (unidentified) neuronal population. Yet from rodent studies there are indications of a close relationship between serotonin and NPY in eating behavior. NPY/AGRP neurons receive serotonin input (Guy et al., 1988; Heisler and Tecott, 1999), and NPY mRNA is increased by administration of exogenous serotonin (Choi et al., 2006). Pharmacological inhibition of serotonin signaling reduced the feeding effect of NPY administration (Bendotti and Samanin, 1987; Grignaschi et al., 1995; Lam et al., 2010).

Interestingly, another area with dense innervation of SERT fibers was the suprachiasmatic nucleus (SCN), the area in the brain were the biological clock resides. This finding on dense innervation is in agreement with literature showing serotonin as well as SERT containing fibers in the SCN in other species (for review: Challet, 2007) and is in line with the important role of serotonin as regulator of the circadian phase. This innervation is also likely to affect feeding behavior and glucose metabolism, which are clearly under the influence of the SCN.

Pharmacological targeting of the serotonin system for the treatment of obesity has received a lot of attention, but the use of pharmacological compounds affecting serotonin signaling has been complicated by unwanted side-effects, such as increased heart rate, hypertension, headaches, and nausea (Lam and Heisler, 2007). The use of specific serotonin reuptake inhibitors for treatment of an array of psychiatric and neurological disorders also shows the importance of serotonin in relation to body weight. Side-effects of specific serotonin reuptake inhibitors include increased appetite and increased body weight (Fava, 2000; Ferguson, 2001; Raeder et al., 2006). Our results on SERT distribution in the human hypothalamus provides an anatomical framework for future investigations regarding the role of the serotonergic system in the human hypothalamus in terms of body weight regulation.

### Conflict of interest statement

The Reviewer, Dr Denise Belsham declares that, despite having collaborated with the author Dr Susanne la Fleur, the review process was handled objectively and no conflict of interest exists. The authors declare that the research was conducted in the absence of any commercial or financial relationships that could be construed as a potential conflict of interest.
